# Chromosomal level genome assemblies of two *Malus* crabapple cultivars Flame and Royalty

**DOI:** 10.1038/s41597-024-03049-x

**Published:** 2024-02-13

**Authors:** Hua Li, Xuyang Zhai, Haixu Peng, You Qing, Yulin Deng, Shijie Zhou, Tairui Bei, Ji Tian, Jie Zhang, Yujing Hu, Xiaoxiao Qin, Yanfen Lu, Yuncong Yao, Sen Wang, Yi Zheng

**Affiliations:** 1 Beijing Key Laboratory for Agriculture Application and New Technique, College of Plant Science and Technology, Beijing University of Agriculture, Beijing, 102206 China; 2 Bioinformatics Center, Beijing University of Agriculture, Beijing, 102206 China

**Keywords:** Plant genetics, Genome informatics

## Abstract

*Malus hybrid* ‘Flame’ and *Malus hybrid* ‘Royalty’ are representative ornamental crabapples, rich in flavonoids and serving as the preferred materials for studying the coloration mechanism. We generated two sets of high-quality chromosome-level and haplotype-resolved genome of ‘Flame’ with sizes of 688.2 Mb and 675.7 Mb, and those of ‘Royalty’ with sizes of 674.1 Mb and 663.6 Mb, all anchored to 17 chromosomes and with a high BUSCO completeness score nearly 99.0%. A total of 47,833 and 47,307 protein-coding genes were annotated in the two haplotype genomes of ‘Flame’, and the numbers of ‘Royalty’ were 46,305 and 46,920 individually. The assembled high-quality genomes offer new resources for studying the origin and adaptive evolution of crabapples and the molecular basis of the accumulation of flavonoids and anthocyanins, facilitating molecular breeding of *Malus* plants.

## Background & Summary

*Malus hybrid* ‘Flame’ (‘Flame’) and *Malus hybrid* ‘Royalty’ (‘Royalty’) are representative ornamental crabapples of the genus *Malus* in the rose family (*Rosaceae*). ‘Flame’ belongs to the ever-green leaf category, with green leaves and white flowers, while ‘Royalty’ belongs to the category of ever-red leaf, with purple-red leaves, flowers and fruits, and the fruit is fetal red^1^. ‘Royalty’ and ‘Flame’ crabapples are rich in flavonoids^2^. In ‘Royalty’, 17, 17, 15 and 9 kinds of flavonoids were detected from the leaves, flowers, peel and flesh respectively, and 15, 17, 11 and 9 types were detected from ‘Flame’ crabapple. And a putative transcription factor, *MdMYB8*, associated with flavonol biosynthesis was discovered by Li *et al*. based on transcriptome analysis of the transcriptomes of the fruit of ‘Flame’ from five continuous developmental stages^3^. Flavonoids are an important class of natural organic compounds with a wide range of biological activities. Previous studies had shown that flavonoids exhibit strong antioxidant activity and possess various pharmacological functions such as antibacterial, anti-inflammatory, anti-tumor, and anti-diabetic effects^4–6^. Therefore, ‘Royalty’ and ‘Flame’ as natural carriers for the synthesis and accumulation of flavonoids have significant utilization value and strong development potential^7,8^. Studying their genomes contributes to research on the pathways of flavonoid accumulation.

In addition, ‘Royalty’ and ‘Flame’ are the preferred materials for studying plant coloration mechanism due to the significant differences in the colors of diverse tissues. For example, as the key anthocyanin regulator, *McMYB10* was identified in leaves and petals of crabapple and relatd to anthocyanin accumulation in ‘Royalty’, a crabapple cultivar with red-colored leaves and flowers^9,10^. Then, the targeted gene *McF3’H*^10^, *McDFR1* promoter^11^ and specific ubiquitin E3 ligases McCOP1-1 and McCOP1-2^12^ of *McMYB10* were found through the investigation of leaf development in the two crabapples, besides that transcription factor McMYB12 promoting the accumulation of proanthocyanidins was discovered^13^. Furthermore, the endogenous *McCHS* gene was proved to be a critical factor during petal coloration by comparing content of flavonoids and anthocyanin of three typical crabapple cultivars with different petal colors^14^. Thus, the genomic data obtained in this study lays the foundation for subsequent investigations using multi-omics analysis strategy to explore the molecular mechanisms of anthocyanin synthesis, which is of great significance for a deep understanding of this important trait of coloring and improving the color breeding of these important ornamental crabapples.

**Fig. 1:**
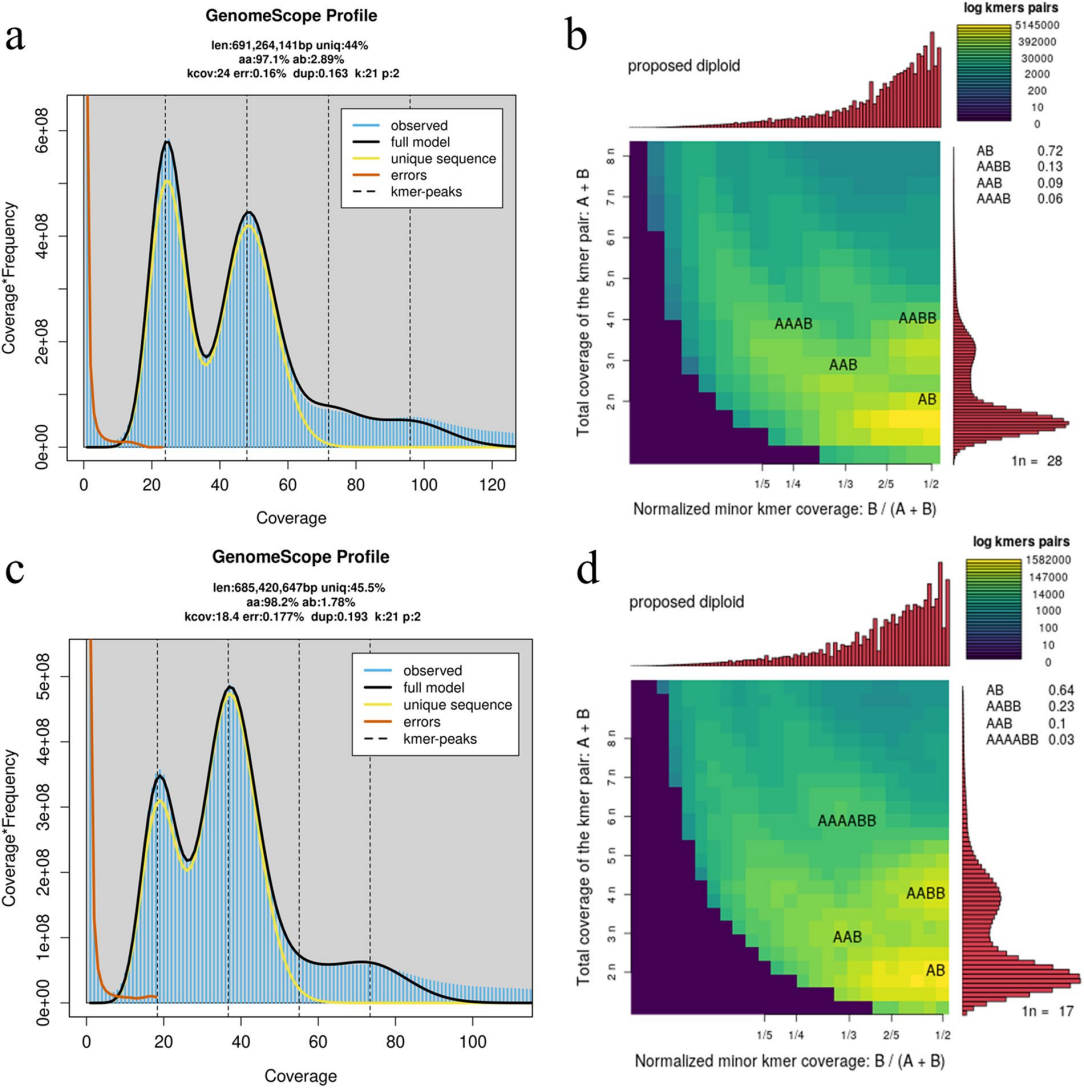
Genome survey results based on K-mer analysis. (**a**) Heterozygosity analysis of ‘Flame’. (**b**) Ploidy analysis of ‘Flame’. (**c**) Heterozygosity analysis of ‘Royalty’. (**d**) Ploidy analysis of ‘Royalty’.

**Fig. 2:**
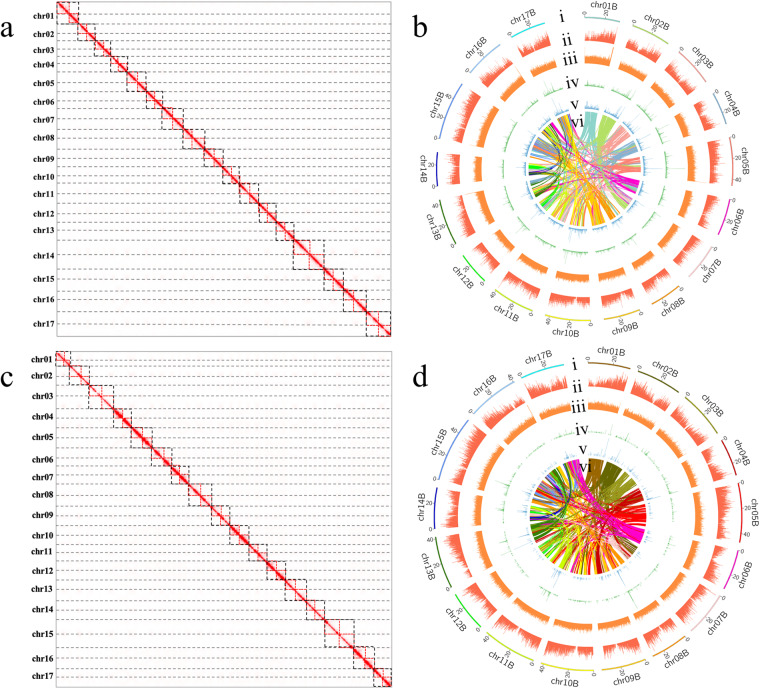
Hi-C interaction analysis and circos map. (**a**) Hi-C interaction heatmap of ‘Flame’. (**b**) The circos map of ‘Flame’. (**c**) Hi-C interaction heatmap of ‘Royalty’. (**d**) The circos map of ‘Royalty’. For the circos map, the tracks from outside to inside are: Chromosome ID and length (i), Density of protein-coding genes (ii), Density of LTR elements (iii), GC content (iv), Density of structural variations (v), Paralog synteny relationships (vi).

**Fig. 3:**
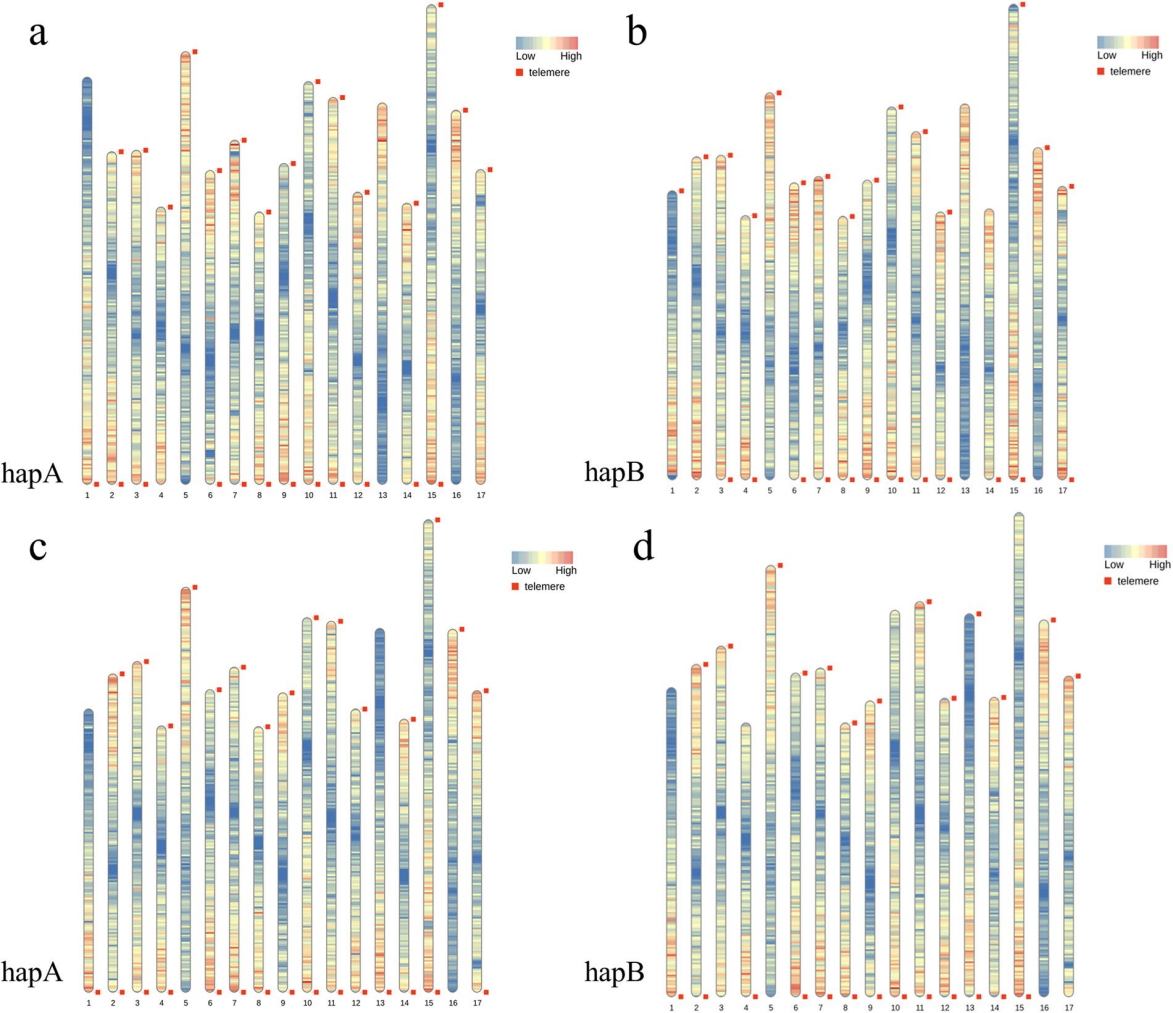
Chromosome telomere location maps of of ‘Flame’-hapA (**a**), ‘Flame’-hapB (**b**), ‘Royalty’-hapA (**c**) and ‘Royalty’-hapB (**d**).

In this study, we present high-quality genomes for *Malus hybrid* ‘Royalty’ and *Malus hybrid* ‘Flame’ using PacBio, Illumina, and Hi-C technologies. The results of k-mer analysis showed that the heterozygosity of ‘Flame’ was 2.89% and the genome size was ~691.2 Mb, while the heterozygosity of ‘Royalty’ was 1.78% and the genome size was ~685.4 Mb, which confirmed that both ‘Royalty’ and ‘Flame’ were highly heterozygous diploids (Fig. 1). The maximum assembled genome of ‘Flame’ (hapA) had a size of 688.2 Mb with a contig N50 of 31.6 Mb and the other was 675.7 Mb with a contig N50 of 35.6 Mb. The two haplotype genomes of ‘Royalty’ were 674.1 Mb with a contig N50 of 23.7 Mb (hapA) and 663.6 Mb with a contig N50 of 28.7 Mb (hapB) (Table 1). The assembled contigs were all further anchored to 17 pseudo-chromosomes, with an anchoring rate of 93.4% in ‘Flame’-hapA, 96.4% in ‘Flame’-hapB, 92.2% in ‘Royalty’-hapA and 95.4% in ‘Royalty’-hapB (Table 1, Fig. 2). The two haplotype genome of ‘Flame’ both had 5 chromosomes assembled into single-ended telomeres, 11 chromosomes assembled into double-ended telomeres, and only 1 chromosome not assembled into telomeres. There were 4 chromosomes assembled into single-ended telomeres and the rest were assembled into double-ended telomeres of ‘Royalty’-hapA, while 8 chromosomes of ‘Royalty’-hapB were equiped with single-ended telomeres and the other 9 chromosomes were with double-ended telomeres (Fig. 3). A total of 47,833 and 47,307 protein-coding genes were identified and almost fully annotated in the two haplotype genomes of ‘Flame’, respectively. All the 46,305 and 46,920 protein-coding genes of the two haplotype genome of ‘Royalty’ in each could be functionally annotated (Tables 2, 3). The quality of the final genomic assembly was assessed to be high gene completeness (‘Royalty’: 98.9% - hapA and 99.0% - hapB; ‘Flame’: 98.9% - hapA and 99.0% - hapB). The assembled high-quality genome of *Malus hybrid* ‘Royalty’ and *Malus hybrid* ‘Flame’ should be a valuable resource for future conservation genomics studies and flavonoid accumulation and anthocyanin synthesis investigations.

**Table 1:** Summary of *Malus hybrid* ‘Flame’ and *Malus hybrid* ‘Royalty’ genome assembly data.

	‘Flame’-hapA	‘Flame’-hapB	‘Royalty’-hapA	‘Royalty’-hapB
Assembled contigs size (Mb)	688.2	675.7	674.1	663.6
Contig N50(Mb)	31.6	35.6	23.7	28.7
Chromosome genome(Mb)	642.9	651.8	628.5	637.4
Unanchored size(Mb)	45.3	23.9	52.8	27.5
Anchoring rate(%)	93.4	96.4	92.2	95.4

**Table 2:** Overview of genome assembly and annotation.

Type	‘Flame’-hapA	‘Flame’-hapB	‘Royalty’-hapA	‘Royalty’-hapB
Gene number	47,833	47,307	46,305	46,920
CDS	223,448	223,709	219,117	222,048
exon	239,248	238,746	236,547	239,522
Three prime UTR	28,863	27,700	29,539	29,720
Five prime UTR	30,660	29,287	32,073	32,011
BUSCOs(%)	99	98.8	98.9	99

**Table 3:** Summary of *Malus hybrid* ‘Flame’ and *Malus hybrid* ‘Royalty’ genome annotations.

	‘Flame’-hapA	‘Flame’-hapB	‘Royalty’-hapA	‘Royalty’-hapB
GenBank-NR	47,708(99.74%)	46,913(99.17%)	46,305(100.00%)	46,920(100.00%)
A. thaliana	38,877(81.28%)	38,009(80.35%)	37,870(81.78%)	38,295(81.62%)
SwissProt	33,459(69.95%)	33,028(69.82%)	32,920(71.09%)	33,295(70.96%)
TrEMBL	45,994(96.16%)	45,319(95.80%)	44,807(96.76%)	45,353(96.66%)
InterPro	31,070(64.96%)	30,098(63.62%)	30,222(65.27%)	30,467(64.93%)
KEGG	20,742(43.36%)	20,705(43.77%)	20,371(43.99%)	20,752(44.23%)
GO	22,116(46.24%)	21,930(46.36%)	21,742(46.95%)	22,034(46.96%)
TF/TR	3,147(6.58%)	3,030(6.40%)	3,095(6.68%)	3,091(6.59%)
PK	1,510(3.16%)	1,606(3.39%)	1,713(3.70%)	1,679(3.58%)

## Methods

### Sample preparation and DNA sequencing

Fresh leaves of *Malus hybrid* ‘Royalty’ and *Malus hybrid* ‘Flame’ were collected, which were located in Beijing University of Agriculture (36°49′N 128°37′E; 575-m altitude), Beijing, China. DNA was isolated from the samples using Cetyltrimethylammonium bromide (CTAB) method and purified by the AMPure PB beads (PacBio 100-265-900) to obtain high-molecular-weight genomic DNA (gDNA, ≥100 ng/μl and ≥10 μg) for subsequent library construction.

For HiFi sequencing, the SMRTbell DNA libraries were constructed using the following steps, according to the PacBio HiFi library construction protocol: (i) gDNA (extracted 10 μg and exceed 40 Kb in majority) target size shearing (15 Kb) using Megaruptor (Diagenode, B06010001) and then concentrated using AMPure® PB Beads (PacBio 100-265-900); (ii) DNA damage repair; (iii) blunt-end ligation with hairpin adapters; and (iv) and enzyme digestion using the SMRTbell® Express Template Prep Kit 2.0 (PacBio, PN 101-853-100); (v) size-selection using the SageELF (Sage Science ELF000) or the BluePippin Size Selection System (Sage Science BLU0001). Subsequently, HiFi sequencing was performed on a PacBio Sequel II platform (PacBio, CA, USA) for 30 hours.

For Hi-C, leaves were fixed in 1% (vol/vol) formaldehyde for library construction. The Hi-C library construction schedule including cell lysis, chromatin digestion, proximity-ligation treatments, DNA recovery and subsequent DNA manipulations were performed according to a previously described method^15^. DpnII was used as the restriction enzyme in chromatin digestion. The Hi-C library was sequenced on the Illumina NovaSeq. 6000 sequencing platform for 150 bp paired-end reads.

### Genome survey and analysis

A total of 36 Gb and 26 Gb high-quality HiFi reads for ‘Flame’ and ‘Royalty’, respectively, were obtained by PacBio Sequel II platform and utilized for genome size and ploidy analysis. The Jellyfish (v2.2.10)^16^ software was performed for k-mer counting of reads from the two genomes, respectively. The reads were cut into 21-base sequences, the total number of 21-mers and the frequency of each 21-mer were counted and the distribution of 21-mers frequencies was plotted. The obtained matrix after 21-mer counting was then used to calculate the haplotype genome size and heterozygosity of ‘Flame’ and ‘Royalty’, as well as the prediction of ploidy, using Genomescope (v2.0)^17^ software. The genome size of ‘Flame’ and ‘Royalty’ were estimated to be 691,264,141 bp and 685,420,647 bp respectively. And the rate of heterozygosity were estimated to be 2.89% and 1.78%, respectively. The K-mer analysis indicated that both ‘Flame’ and ‘Royalty’ were highly heterozygous diploids (Fig. 1).

### Genome assembly

Contigs were *de novo* assembled from PacBio HiFi reads to generate a phased assembly graph and then HiC reads were ultilized to link unitigs that share mapped fragments by hifiasm (v0.16.1)^18^ with parameters (–hom-cov 34–n-weight 6 -s 0.45 -O 2). Following that, contigs were anchored into 34 chromosomes in total using the software Juicer^19^ and the 3D-DNA^20^ (-m haploid -r 0) based on Hi-C interaction data (‘Flame’: 60 Gb, ~100×; ‘Royalty’: 80 Gb, ~133×) (Fig. 2). Subsequently, the assembled genome was manually corrected with JucieBox^21^, including correcting chromosome boundaries, rejoining misjoins, and addressing inversions and translocations, and the final genome was generated using agp2fa mode of RagTag^22^ based on AGP format file recording contigs of each chromosome. The total length of two chromosome-level haplotype-resolved genomes of ‘Flame’ was 642.9 Mb (hapA) with a contig N50 of 31.6 Mb and 651.8 Mb (hapB) with a contig N50 of 35.6 Mb, of which of ‘Royalty’ was 628.5 Mb (hapA) with a contig N50 of 23.7 Mb and 637.4 Mb (hapB) with a contig N50 of 28.7 Mb, achieving anchoring rate of all haploid genomes higher than 92% (Table 1).

The telomere sequences were detected with the software TRF^23^ and most of the chromosomes are assembled to telomeres. For examples, a total of 5 chromosomes assembled into single-ended telomeres, 11 chromosomes assembled into double-ended telomeres, and only 1 chromosome not assembled into telomeres of ‘Flame’, while 4 chromosomes assembled into single-ended telomeres and 13 chromosomes assembled into double-ended telomeres of ‘Royalty’-hapA, confirmed the high genomic integrity and continuity of the assembled genomes (Fig. 3).

### Genome annotation

Repeat sequences were annotated using de-novo approaches, by constructing a database of repeat sequences using the software RepeatModeler (v1.0.11)^24^ with setting parameters (-database -pa 5). Subsequently, the constructed database was imported to RepeatMasker (v4.1.2)^25^ to identify transposons or low-complexity repeats in the DNA sequences, and then the TRF (v4.09)^23^ was used to identify tandem repeats. It had been found that both of ‘Royalty’ and ‘Flame’ genomes were highly repetitive, of which 64.76% were repetitive sequences in ‘Flame’, and the major portion of the repetitive sequences was the retransposon LTR at a percentage of about 37.74%. In ‘Royalty’, 64.59% were repetitive sequences, and the repetitive sequences that accounted for the most part of the repetitive sequences were also LTRs about 34.62%.

To annotate a complete and accurate gene structure, a strategy incorporating transcriptome, protein-based homology, and *ab initio* prediction was employed^26^. For transcript-based prediction, two sets of published transcriptome data (‘Flame’ was assisted by BioProject PRJNA546094^27^ and ‘Royalty’ was assisted by BioProject PRJNA546107^28^) were mapped to the assembled genomes, respectively, by HISAT2 (v2.2)^29^. The mapped reads were assembled by StringTie (v1.3)^30^ to retain the longest transcripts as EST evidence. As for protein-based homology, the protein sequences of sequenced apple genomes of ‘Golden Delicious’ (*Malus domestica* cv. Golden Delicious), ‘Hanfu’ (*Malus domestica* cv. Hanfu), ‘Gala’ (*Malus domestica* cv. Gala), European wild apple (*Malus sylvestris*) and wild apple (*Malus sieversii*) were utilized to perform homology prediction by Exonerate^31^. For the *ab initio* prediction, the assemblies were hard masked according to the repeat annotation, and then Augustus (v3.4)^32^ and BRAKER2^33^ were performed to train a gene prediction model based on the transcripts. At last, protein coding genes were predicted using BRAKER2 with the trained model. Finally, the predictions generated by the above methods were integrated to generate the final of the annotation file by using the Maker (v3.1)^34^. Comparison of the protein-coding genes with single-copy homologous conserved gene databases using BUSCO (v4.1)^35^ analysis showed that the two sets of haplotype genome sequences of ‘Flame’ contained complete homologous conserved genes in about 99.0% and 98.8% of plants, and those of ‘Royalty’ were about 98.9% and 99.0% respectively (Table 2).

The functional annotation was performed following a standard workflow based on above annotated protein-coding genes: (i) Diamond (v2.0)^36^ was run with an E-value threshold of 1e-4 against GenBank-NR^37^, Swiss-Prot^38^, TrEMBL^39^ and the Arabidopsis protein database^40^; (ii) InterProScan (v5.59)^41,42^ was performed to identify functional protein structural domains against the InterPro^42^ database; (iii) aligned results from the GenBank-NR database were combined with identified functional domains of InterPro proteins for GO (the gene Ontology Consortium)^43^ annotation using the Blast2GO (v2.2)^44^ program; (iv) the annotation results of SwissProt, TrEMBL, and Arabidopsis protein database were combined with AHRD (v3.3) program; (v) the Kyoto Encyclopedia of Genes and Genomes (KEGG)^45^ database was also consulted for KEGG functional annotations in Blast2GO (v2.2); (vi) Prediction of transcription factors (TF), transcriptional regulators (TR) and protein kinases (PK) for protein-coding genes using iTAK^46^ software.

The final annotation results showed that the hapA and hapB genomes of ‘Flame’ contain 47,833 and 47,307 genes respectively. For ‘Royalty’, 46,305 genes were annotated in the hapA genome and 46,920 in the hapB genome (Table 2). For the functional annotations of ‘Falme’, the protein-coding genes were compared with the GenBank-NR, SwissProt, *Arabidopsis* protein database, and TrEMBL databases, and of each was annotated 94,621, 66,487, 76,886, and 91,313 genes, respectively. A total of 44,046 genes were matched to GO database and 41,447 genes were linked with pathway annotations. 6.58% of genes were indentified as TFs/TRs and 3.16% were labeled as PK. As for ‘Royalty’, there were 93,225, 66,215, 76,165 and 90,160 genes matched with the GenBank-NR, SwissProt, *Arabidopsis* protein database, and TrEMBL databases, separately. Additionally, 43,776 and 41,123 genes were annotated by GO and KEGG in each. The total number of predicted transcription factors and transcriptional regulators was similar to the Flame’s, but the identified protein kinases were 276 over than Flame’s, counting for 3.58% of total genes (Table 3).

## Data Records

The raw data (PacBio HiFi reads, and Hi-C sequencing reads) used for genome assembly were deposited in the NCBI database under BioProject accession PRJNA1026659^47^. The chromosomal assembly and dataset of gene annotation have been deposited at Figshare (10.6084/m9.figshare.24276916)^48^. The assembled diploid genome of ‘Flame’ was deposited in GenBank database (accession number: GCA_036218565.1^49^ for hapA and GCA_036220445.1^50^ for hapB). The assembly genome files of ‘Royalty’ were stored under the accession GCA_036320615.1 (hapA)^51^ and GCA_036320635.1 (hapB)^52^, respectively.

## Technical Validation

Firstly, the Hi-C heatmap exhibits the accuracy of genome assembly, with relatively independent Hi-C signals observed between the 17 pseudo-chromosomes (Fig. 2). Furthermore, the completeness of the genomes was evaluated using the BUSCO pipeline based on the embryophyta_odb10 database. BUSCO assessment of the final hapA genome of ‘Flame’ found that 99.0% of the 1,614 highly conserved orthologs were present as complete genes, including 61.3% single-copy BUSCOs and 37.7% duplicated BUSCOs and the completeness score of hapB genome was 98.8% with 62.1% single-copy BUSCOs. The final hapA genome of ‘Royalty’ found that 98.8% of the 1,614 highly conserved orthologs were present as complete genes, including 63.3% single-copy BUSCOs and 35.5% duplicated BUSCOs, and the hapB genome had a similar performance. Also, It had been showed by chromosome telomere location map that the assembled genome is assembled to telomeres except for the 13 chromosome of ‘Flame’, and most of the chromosomes were assembled into double-ended telomeres (Fig. 3).

## Data Availability

There is no custom code was used during this study. All software and pipelines were executed according to the manual and protocols of the published bioinformatics tools. The version and code/parameters of software have been detailed and described in Methods.
